# Classic polyarteritis nodosa associated with hepatitis C virus infection: a case report

**DOI:** 10.1186/1752-1947-6-305

**Published:** 2012-09-14

**Authors:** Damith Rodrigo, Ruwan Perera, Janaka de Silva

**Affiliations:** 1 University Medical Unit, Colombo North Teaching Hospital, Ragama, Sri Lanka

**Keywords:** Hepatitis C virus, Vasculitis, Polyarteritis nodosa

## Abstract

**Introduction:**

Hepatitis C virus has been under-recognized as an etiologic factor for polyarteritis nodosa and the presence of hepatitis C antigenemia in patients with polyarteritis nodosa has been reported as insignificant. In the literature hepatitis C virus-associated polyarteritis nodosa is a rare and controversial entity.

**Case presentation:**

A 34-year-old Sri Lankan Tamil man presented to our facility with a two-week history of slow-resolving pneumonia of the right mid and lower zones. On physical examination he had panniculitic type tender skin nodules with background livedo reticularis. A skin biopsy was suggestive of a small and medium vessel vasculitis compatible with polyarteritis nodosa. He was tested positive for hepatitis C antibodies. A serum cryoglobulin test was negative but perinuclear antineutrophilic cytoplasmic antibody test was positive. Serum complement levels were reduced. He was diagnosed as having classic polyarteritis nodosa associated with hepatitis C infection. He later developed left-sided radiculopathy involving both upper and lower limbs and an ischemic cardiac event. His hepatitis C infection was managed with polyethylene glycol-interferon 2α combined with oral ribavirin. Simultaneously, his classic polyarteritis nodosa was treated with prednisolone and cyclophosphamide. He made a good recovery.

**Conclusions:**

Hepatitis C virus infection is capable of inducing a fulminant form of vasculitis in the form of polyarteritis nodosa. It may be easily confused early in its course with mixed cryoglobulinemia, which is commonly known to be associated with hepatitis C virus. Awareness of hepatitis C virus-related polyarteritis nodosa helps in diagnosing the condition early so combined immunosuppressive and antiviral treatment can be started as soon as possible.

## Introduction

Hepatitis B virus (HBV) is a well-known etiologic factor of polyarteritis nodosa (PAN), whereas hepatitis C virus (HCV) is commonly associated with cryoglobulinemic vasculitis [1,2]. HCV has been under-recognized as an etiologic factor for PAN and the presence of hepatitis C antigenemia in patients with PAN has been reported as being insignificant [3,4]. In the literature HCV-associated PAN is a rare and controversial entity. Here, we report a case of PAN related to HCV infection.

## Case presentation

**Figure 1:**
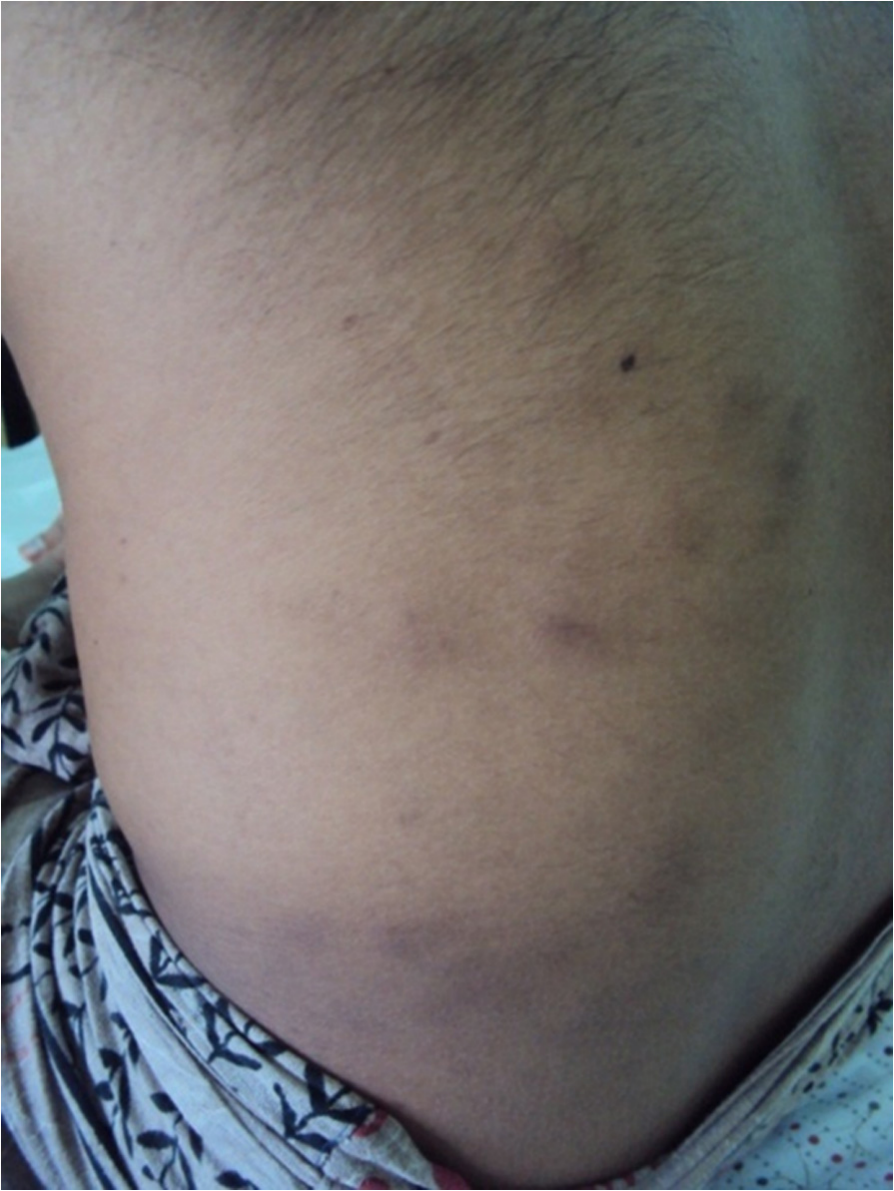
Panniculitic type skin rash with tender hard nodules.

A 34-year-old, unmarried, previously healthy Sri Lankan Tamil man was transferred from a private hospital with a two-week history of slow-resolving pneumonia of the right mid and lower zones. He had recently returned to Sri Lanka after working abroad. He complained of malaise, loss of appetite and significant loss of weight. On physical examination he was found to have generalized tender lymphadenopathy, and panniculitic type tender, hard skin nodules were scattered all over his body with background livedo reticularis and a few ulcers (Figure 1). A respiratory system examination showed right mid and lower zone bronchial breathing.

A chest X-ray showed a resolving right-sided mid and lower zone consolidation. His hemoglobin level was 10.6g/dL with a total leukocyte count of 4.89×10^9^ cells/L and platelet count of 172×10^9^ cells/L. Renal function test results were within normal limits. His liver profile showed an aspartate aminotransferase level of 71U/L, alanine aminotransferase level of 23U/L and serum albumin level of 2.8g/dL. His erythrocyte sedimentation rate (ESR) was 88mm in the first hour and C-reactive protein level was 32.8mg/dL (normal <0.8mg/dL). Ultrasound of our patient’s abdomen showed hepatosplenomegaly.

Hepatitis serology was performed and he was found to be positive for hepatitis C antibodies (anti-HCV). Initial HCV polymerase chain reaction tests showed 6000 viral copies/mL. He was tested negative for hepatitis B surface antigen and anti-HIV. Tests for rheumatoid factor and serum cryoglobulin were negative, but a test for perinuclear antineutrophilic cytoplasmic antibody (pANCA) was positive. His serum complement levels were reduced with C3=56mg/dL (normal 83 to 111) and C4=3mg/dL (normal 12 to 36).

**Figure 2:**
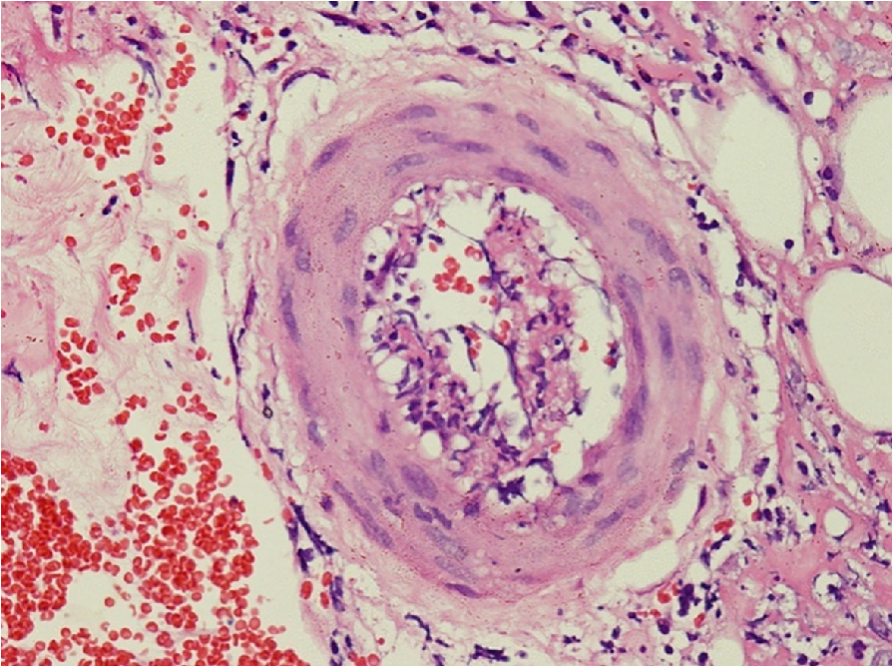
Skin biopsy showing perivascular lymphocytic and neutrophilic infiltration with fibrinoid necrosis of vessel wall with leukocytoclasia and red cell extravasations.

While in the ward our patient developed a lower motor neuron type, left-sided, painful weakness in both upper and lower limbs. Nerve conduction tests confirmed a left-sided radiculopathy. He underwent skin biopsy for the panniculitis, and histology results showed a perivascular lymphocytic and neutrophilic infiltration with fibrinoid necrosis of the vessel wall with leukocytoclasia and red cell extravasations. The features were suggestive of a small and medium vessel vasculitis compatible with polyarteritis nodosa (Figure 2).

Our patient was discharged with a plan to readmit in one week to start antiviral treatment for hepatitis C. During his second admission he complained of a left-sided ischemic type chest pain with autonomic symptoms, and was found to have high blood pressure of 210/160mmHg. His electrocardiogram showed acute anterolateral ischemia. A troponin I test was negative, but a two-dimensional echocardiogram showed global hypokinesia with dilated cardiomyopathy. Considering his young age and in the absence of coronary artery disease risk factors except for smoking, the possibility that the acute coronary event was related to the vasculitis process was considered.

Our patient was diagnosed as having classic polyarteritis nodosa (cPAN) associated with hepatitis C infection. He fulfilled the American College of Rheumatology (ACR) criteria and the Chapel Hill Consensus Conference (CHCC) nomenclature of systemic vasculitis for the diagnosis of classic polyarteritis nodosa with cardiac, neurological and cutaneous involvement [5,6].

After being treated for the acute coronary event he was started on treatment for the underlying conditions. His hepatitis C infection was treated with polyethylene glycol-interferon 2α 180μg weekly combined with oral ribavirin 400mg three times a day. Simultaneously, he was started on prednisolone 10mg twice a day and oral cyclophosphamide 25mg once a day for classic polyarteritis nodosa.

He was reviewed after six weeks and was found to have made a good recovery with healed skin lesions and improvement of motor weakness. His blood pressure was 110/70mmHg and his ESR was normal at 12mm in the first hour. We have now been following him up in our clinic for nearly six months without any relapse.

## Discussion

HBV was once the cause of up to 30% of PAN cases [7]. Widespread use of the hepatitis B vaccine has significantly decreased the incidence of HBV-PAN, which is now estimated to account for less than 8% of all PAN cases [8]. Mixed cryoglobulinemia was previously the most widely known extrahepatic vasculitis manifestation of hepatitis C virus. In a recent study among a cohort of 161 patients with HCV-related vasculitis, 31 (19.3%) were diagnosed as having PAN, and compared with HCV-mixed cryoglobulinemia (MC) vasculitis, HCV-PAN displayed a more severe and acute clinical presentation and higher rate of clinical remission [9]. Similar results were shown in another study by Cacoub *et al*. [10]. Corticosteroids are the cornerstone of treatment for PAN, and are combined with cyclophosphamide to control its severe life-threatening complications. There is also a recent report of a life-threatening hepatitis C virus-associated polyarteritis nodosa successfully treated with rituximab [11].

## Conclusions

In summary, we would like to highlight the possibility of HCV infection being capable of inducing a fulminant type of vasculitis in the form of PAN. It may be easily confused early in its course with mixed cryoglobulinemia, which is commonly known to be associated with hepatitis C virus. Awareness of HCV-related PAN helps in diagnosing the condition early and ensuring combined immunosuppressive and antiviral treatment is started as soon as possible.

## Consent

Written informed consent was obtained from the patient for publication of this case report and any accompanying images. A copy of the written consent is available for review by the Editor in Chief of this journal.

## Competing interests

The authors declare that they have no competing interests.

## Authors’ contribution

DR, RP and HdeS were involved actively in the management of the patient. DR drafted the manuscript. All the others provided valuable inputs and guidance during the preparation of the manuscript. All authors read and approved the final manuscript.
